# Cerebral Infarctions After Transcatheter Aortic Valve Replacement of Horizontal Aorta Versus Non-Horizontal Aorta

**DOI:** 10.3390/jcm15176715

**Published:** 2026-08-29

**Authors:** Hanyi Dai, Dao Zhou, Wenjing Sheng, Rongrong Zheng, Jun Chen, Abuduwufuer Yidilisi, Xianbao Liu, Lihan Wang

**Affiliations:** 1 Department of Cardiology, The Second Affiliated Hospital, School of Medicine, Zhejiang University, Hangzhou 310009, China; 22018285@zju.edu.cn (H.D.); zhoud@zju.edu.cn (D.Z.); 22318381@zju.edu.cn (W.S.); chen_jun@zju.edu.cn (J.C.); 21918225@zju.edu.cn (A.Y.); 2 State Key Laboratory of Transvascular Implantation Devices, Hangzhou 310009, China; 3 Heart Regeneration and Repair Key Laboratory of Zhejiang Province, Hangzhou 310009, China; 4 Transvascular Implantation Devices Research Institute, Hangzhou 310053, China; 5 Hangzhou Emergency Medical Center of Zhejiang Province, Hangzhou 310021, China; y216180149@zju.edu.cn; 6 Binjiang Institute, Zhejiang University, Hangzhou 310052, China

**Keywords:** transcatheter aortic valve replacement, aortic stenosis, cerebral ischemic infarctions, horizontal aorta

## Abstract

**Background/Objectives**: Horizontal aorta, characterized by a high aortic angle, represents a challenging anatomical feature during transcatheter aortic valve replacement (TAVR), but its association with cerebral ischemic injury remains unclear. This study investigated the association between horizontal aorta and diffusion-weighted magnetic resonance imaging (DW-MRI)-detected cerebral infarctions after TAVR. **Methods**: We retrospectively enrolled 411 consecutive patients with severe aortic stenosis undergoing transfemoral self-expanding TAVR between March 2017 and May 2022. Aortic angle was defined as the angle between the aortic annular plane and the horizontal plane on preprocedural computed tomography, and horizontal aorta was defined as an aortic angle > 60°. Patients were categorized into horizontal aorta and non-horizontal aorta groups. The incidence, number, and volume of new DW-MRI lesions were compared between groups. **Results**: The median age was 74.0 years (interquartile range [IQR]: 69.0 to 79.0); 54.5% were male, and the median Society of Thoracic Surgeons score was 3.83% (IQR: 2.29 to 6.39). Horizontal aorta was present in 69 patients (16.8%). Procedural complications were infrequent and comparable between groups. Although the incidence of new cerebral infarctions was similar between groups (89.9% vs. 83.0%; *p* = 0.157), patients with horizontal aorta had more cerebral infarction lesions (median, 4.0 vs. 3.0; *p* = 0.031), larger total lesion volume (320.0 vs. 180.0 mm^3^; *p* = 0.017), and larger lesion volume per infarction (70.0 vs. 51.7 mm^3^; *p* = 0.036). Multivariable analysis identified horizontal aorta as an independent predictor of the number of post-TAVR cerebral infarctions. **Conclusions**: Horizontal aorta is relatively common among patients undergoing TAVR and is independently associated with a greater burden of DW-MRI-detected subclinical cerebral ischemic injury.

## 1. Introduction

Sufficient evidence from clinical trials has shown the noninferiority of transcatheter aortic valve replacement (TAVR) over standard surgical aortic valve replacement (SAVR) for the treatment of severe symptomatic aortic stenosis (AS), even in the low surgical-risk spectrum [1,2,3,4]. Despite the promising outcomes in real-world practice, cerebrovascular events remain among the most devastating complications following TAVR [3,4,5]. The reported incidence of clinically overt ischemic stroke within 30 days ranges from 1.4% to 4.3%, depending on study populations and endpoint definitions [5]. Although relatively uncommon, periprocedural overt stroke is associated with substantial disability, increased mortality, and poorer long-term outcomes [5,6,7]. In contrast, diffusion-weighted magnetic resonance imaging (DW-MRI) detects new cerebral ischemic lesions in 68–93% of patients after TAVR. Although most lesions are clinically silent, accumulating evidence suggests that they may contribute to impaired cognitive function, reduced physical performance, and diminished quality of life [8,9,10,11].

The aortic angle, defined as the angle between the horizontal plane and the aortic annular plane on multidetector computed tomography (MDCT), is an important anatomical characteristic for TAVR planning [12,13]. A large aortic angle, commonly referred to as a horizontal aorta, is not uncommon among patients undergoing TAVR, although its reported prevalence varies considerably depending on the aortic angle threshold used for its definition [14]. A horizontal aorta may increase procedural complexity by making device advancement and coaxial prosthesis alignment more challenging, particularly when self-expanding valves are used [13,14,15]. Consequently, patients with an aortic angle > 70° have been excluded from several prospective trials of self-expanding TAVR devices [15]. Previous studies have also demonstrated that a larger aortic angle is associated with lower device success and less favorable procedural outcomes [16]. However, whether horizontal aorta influences cerebral ischemic injury after TAVR has not been investigated. Therefore, this study aimed to compare DW-MRI-detected cerebral infarctions after transfemoral self-expanding TAVR between patients with horizontal aorta and those with non-horizontal aorta, and to evaluate whether horizontal aorta is associated with a greater burden of postprocedural cerebral ischemic injury.

## 2. Materials and Methods

### 2.1. Study Population

Consecutive patients admitted to a single tertiary institution between March 2017 and May 2022 were enrolled. Related data, including baseline information, perioperative characteristics, and clinical outcomes, were collected prospectively in the TORCH registry (Transcatheter Aortic Valve Replacement Single Center Registry in Chinese Population, NCT02803294) database. The TORCH registry was prospectively registered at ClinicalTrials.gov (https://clinicaltrials.gov/study/NCT02803294) (accessed on 6 August 2026). The study was first submitted on 7 June 2016, and first posted on 16 June 2016. The present study is a retrospective secondary analysis of prospectively collected registry data. The study protocol was approved by the local human ethics committee and compliant with the principles expressed in the Declaration of Helsinki. All enrolled patients were informed about the details of the TAVR procedure and signed informed consent forms for participating in the registry and the use of anonymous data.

**Figure 1:**
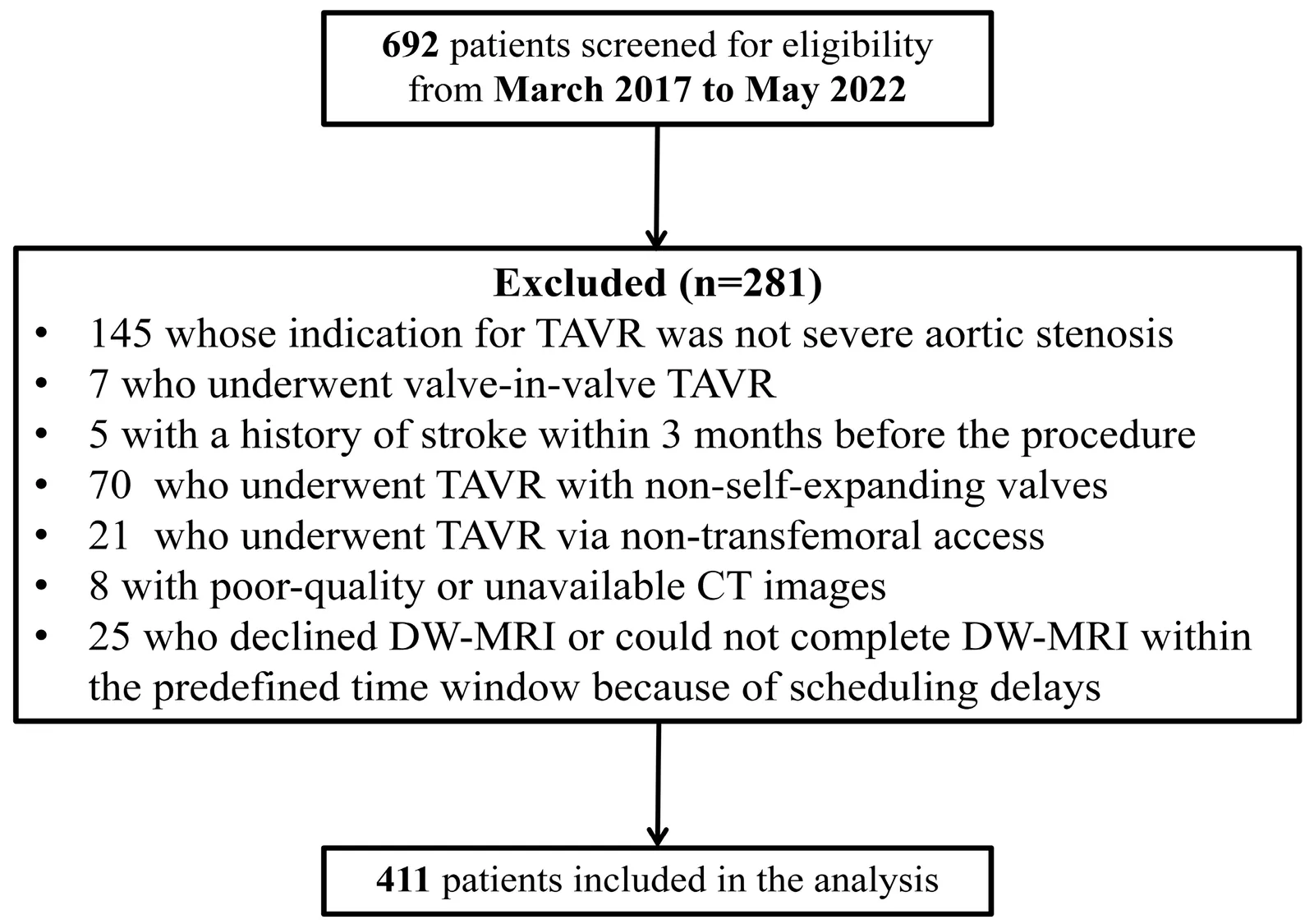
Flow diagram of patient screening, exclusions, and final inclusion. CT, computed tomography; DW-MRI, diffusion-weighted magnetic resonance imaging; TAVR, transcatheter aortic valve replacement.

Detailed inclusion and exclusion criteria were designed before this analysis. From March 2017 to May 2022, a total of 692 consecutive patients undergoing TAVR at our institution were screened for eligibility. Patients were excluded if the indication for TAVR was not severe aortic stenosis (*n* = 145), they underwent valve-in-valve TAVR (*n* = 7), had a history of stroke within 3 months before the procedure (*n* = 5), received a non-self-expanding valve (*n* = 70), underwent TAVR via a non-transfemoral approach (*n* = 21), had poor-quality or unavailable preprocedural CT images (*n* = 8), or declined DW-MRI or could not complete DW-MRI within the predefined time window because of scheduling delays (*n* = 25). Finally, 411 patients were included in the analysis. The patient selection process is shown in Figure 1.

### 2.2. MDCT Assessment and TAVR Procedure

**Figure 2:**
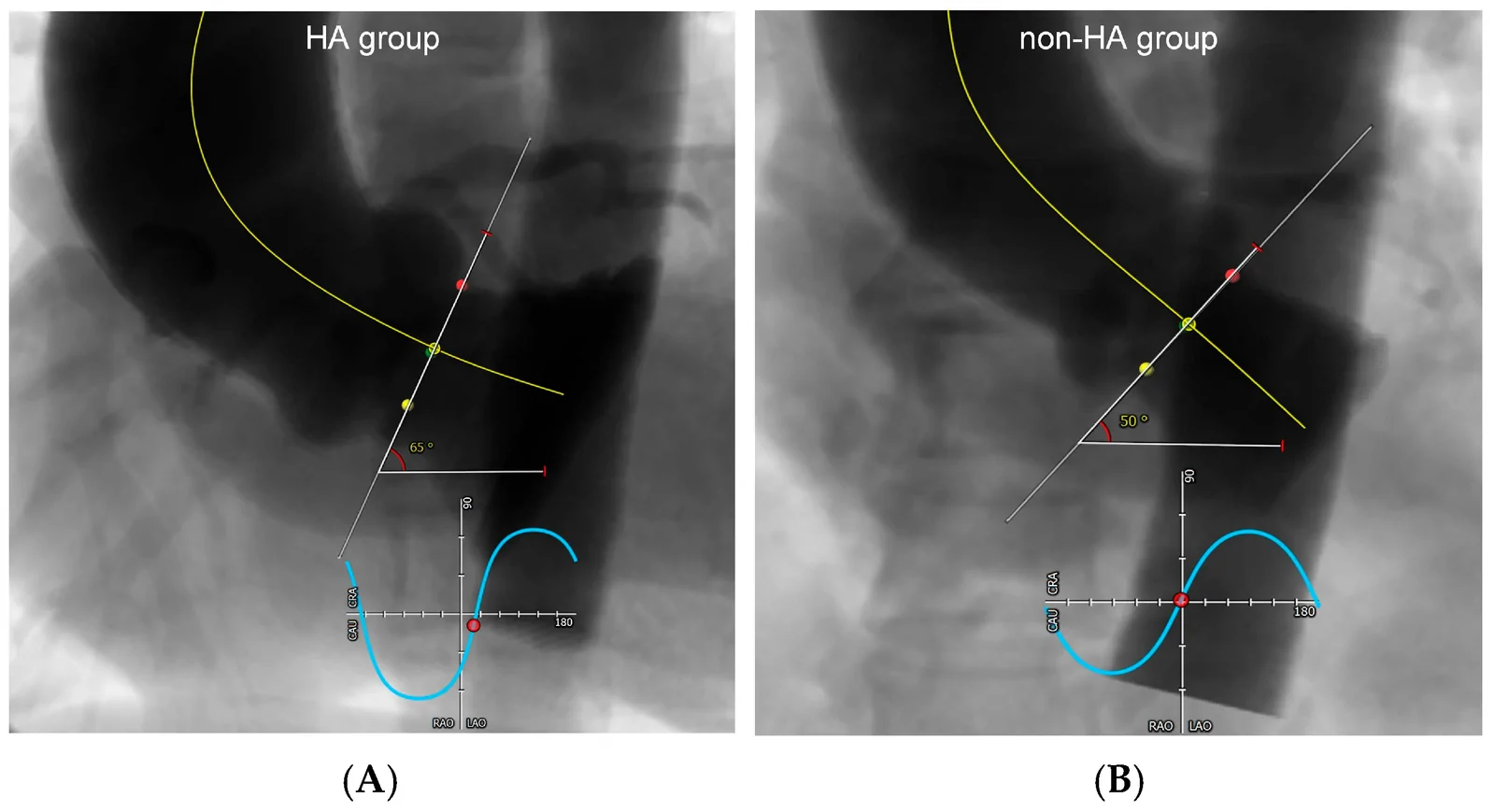
Measurement of the aortic angle on reconstructed multidetector computed tomography images. (**A**,**B**) Example of horizontal aorta and non-horizontal aorta. The oblique gray line represents the projection of the aortic annular plane, and the horizontal gray line represents the horizontal reference plane. The red arc and the corresponding numerical value indicate the measured aortic angle. The yellow curved line represents the reconstructed aortic centerline. The colored dots are software-defined reference points used for image reconstruction and angle measurement, whereas the blue curve and coordinate axes indicate the orientation of the reconstructed image. HA, horizontal aorta.

All TAVR procedures were performed in the hybrid operating room as described before. Pre-procedural cardiac MDCT was routinely performed using Philips Brilliance iCT 256 (Philips Corporation, Amsterdam, The Netherlands) or GE revolution CT (GE Healthcare, Chicago, IL, USA) with collimation of 0.6 or 0.8 mm, 100 or 120 kV for imaging. MDCT images were analyzed by using a 3mensio workstation (version 10.3, 3mensio Medical Imaging BV, Bilthoven, The Netherlands) for analyzing procedural-related aortic root anatomic structures. Aortic angle was defined as the angle between the plane of the aortic annulus and an ideal horizontal plane in a coronal projection and was defined as horizontal aorta if aortic angle > 60° for subsequent analyses (Figure 2).

Before the procedure, all catheters and guidewires were thoroughly flushed with heparinized saline to prevent thrombus formation. Intravenous unfractionated heparin was administered during TAVR, with the activated clotting time maintained between 250 and 300 s and monitored every 30 min. All implanted prostheses in the study were self-expanding valves: early-generation devices (the Medtronic CoreValve System [Medtronic, Minneapolis, MN, USA], VenusA Valve [Venus Medtech, Hangzhou, China], VitaFlow I [Microport, Shanghai, China], and TaurusOne Valve [Peijia Medical, Suzhou, China] and newer-generation devices (VenusA-Plus [Venus Medtech, Hangzhou, China], VitaFlow II [Microport, Shanghai, China] and TaurusOne Elite [Peijia Medical, Suzhou, China]). Predilatation was routinely performed to assess the supra-annular structure assessment method in our center. Preprocedural loading dose of dual antiplatelet therapy, including acetylsalicylic acid 300 mg and clopidogrel 300 mg, was not mandatory. After the procedure, patients with an indication for oral anticoagulation received anticoagulant therapy according to their clinical indications, whereas those without an indication for anticoagulation received dual antiplatelet therapy for 3–6 months followed by lifelong single antiplatelet therapy.

### 2.3. Echocardiographic Assessment

Transthoracic echocardiography was performed before and after TAVR by experienced echocardiographers according to standard clinical practice. Left ventricular ejection fraction was assessed using the biplane Simpson method whenever feasible. Aortic stenosis severity was evaluated based on peak aortic valve velocity, mean transvalvular pressure gradient, and aortic valve area calculated using the continuity equation. The severity of mitral and tricuspid regurgitation was assessed according to guideline-recommended criteria. All echocardiographic parameters used in the present analysis were obtained from preprocedural and postprocedural echocardiographic examinations.

### 2.4. DW-MRI Assessment

All eligible patients had high-quality DW-MRI images obtained using the 1.5 T or 3.0 T imaging system (GE Signa, GE Healthcare, Milwaukee, WI, USA) at baseline and within 7 days after TAVR to detect cerebral infarctions. Two experienced physicians blinded to the patient clinical features checked the quality of cerebral MRI images and then interpreted them on the MRIcron software (version 1.0.2019, NeuroImaging Tools and Resources Collaboratory, Columbia, SC, USA). Moreover, difficult and complex images were reviewed with a senior radiologist together. DW-MRI was conducted with a spin-echo echo-planar pulse sequence (1.5-Tesla: repetition time/echo time = 2921/78 ms, matrix = 128 × 256; slice thickness = 5 mm; intersection gap = 1 mm; total acquisition time = 21.4 s; 3-Tesla: repetition time/echo time = 3866/47 ms, matrix = 128 × 256) with diffusion sensitization b-values of 0 and 1000 s/mm^2^. Additionally, images were obtained in 5 mm slices with a 1 mm intersection gap in both 1.5-Tesla and 3-Tesla systems. New ischemic cerebral lesions were defined as a focal hyperintense area detected in the DWI sequence and confirmed by apparent diffusion coefficient (ADC) mapping to rule out a shine-through artifact. DWI and ADC maps of patients were reviewed simultaneously to detect and localize cerebral infarctions before and after procedure.

### 2.5. Outcomes and Definitions

The primary imaging outcomes were the incidence, number, and volume of newly detected cerebral ischemic lesions on postprocedural DW-MRI. New cerebral ischemic lesions were defined as newly appearing focal hyperintense lesions on DW-MRI after TAVR, confirmed by apparent diffusion coefficient mapping to exclude shine-through artifacts. Lesion burden was assessed according to the number of new lesions per patient, total lesion volume per patient, maximal lesion volume per patient, and lesion volume per lesion.

The secondary clinical endpoints included all-cause mortality, stroke, myocardial infarction, life-threatening bleeding, permanent pacemaker implantation, coronary obstruction, annular rupture, aortic dissection, conversion to surgical aortic valve replacement, and other adverse events occurring within 30 days. Procedural and clinical outcomes were defined according to standardized Valve Academic Research Consortium criteria where applicable.

### 2.6. Statistical Analysis

Predefined stratified analyses were performed on SPSS software package, version 25 (IBM, Armonk, NY, USA) for the comparison of ischemic cerebral infarctions after TAVR. Mean ± standard deviation, median (IQR) as well as count (percentage) were used to describe the characteristics of the study population as appropriate. Student’s *t*-test and Wilcoxon rank-sum test were computed for the comparison of continuous variables while Chi-square test for categorical variables between two groups. Linear regression analyses were carried out to identify the effect of clinical variables on the number of ischemic cerebral infarctions complicating TAVR. Variables with a *p* < 0.10 in univariate analyses were kept in the final model. Statistical significance was set at the 0.05 level (two-tailed).

## 3. Results

### 3.1. Baseline Characteristics

The study population consisted of 411 patients (69 with horizontal aorta and 342 without) undergoing transfemoral TAVR during the study period. The median age of the enrolled patients was 74 years and 224 patients (54.5%) were male. The overall study population had a median STS score of 3.83 (IQR: 2.29 to 6.39). Baseline characteristics of patients with and without horizontal aorta are compared in Table 1. The median aortic angle was 65.0° (IQR: 62.0–69.0) in the horizontal aorta group and 49.0° (IQR: 43.0–53.0) in the non-horizontal aorta group. Patients in the horizontal aorta group also had a greater BMI and were more frequently classified as NYHA class III/IV than those in the non-horizontal aorta group (88.4% vs. 76.9%; *p* = 0.033). Bicuspid aortic valve (BAV) was present in 68.1% of the horizontal aorta group and 56.4% of the non-horizontal aorta group (*p* = 0.072). There were no significant differences in the frequency of comorbidities between the two groups, except that hypertension was more frequent in patients with horizontal aorta (68.1% vs. 50.3%, *p* = 0.007).

As for echocardiographic characteristics, the mean left ventricular ejection fraction of the cohort was 60.6%, the mean aortic valve gradient was 52.0 mmHg, and the peak velocity was 4.75 m/s. Overall, the median annulus perimeter derived diameter was 24.3 mm. The dimensions of the sinuses of Valsalva were larger in patients with horizontal aorta. The diameter (33.0 mm [IQR: 30.4 to 34.7 mm] vs. 29.8 mm [IQR: 26.9 to 32.2 mm]; *p* < 0.001) and the height (22.1 mm [IQR: 19.7 to 25.2 mm] vs. 21.6 mm [IQR: 19.1 to 23.6 mm]; *p* = 0.007) of the STJ in horizontal aorta group were greater than those in non-horizontal aorta group. Likewise, it was larger in the diameter of ascending aorta at the level of 40 mm above annulus in horizontal aorta group (40.0 mm [IQR: 37.9 to 42.8 mm] vs. 36.6 mm [IQR: 33.8 to 40.2 mm]; *p* < 0.001). There were no differences in the bilateral coronary artery ostium height between two groups.

### 3.2. Procedural Details and Early Clinical Outcomes

The procedural characteristics and outcomes are summarized in Table 2. All patients underwent self-expanding TAVR via the transfemoral approach. Balloon pre-dilation was routinely performed. More specifically, the majority of the patients were implanted with VenusA and VitaFlow valves. There were no differences between the two groups in the frequency of pre-dilation (98.6% vs. 97.1%; *p* = 0.168) and post-dilatation (65.2% vs. 67.0%; *p* = 0.780). The incidence of peri-TAVR outcomes was generally low and comparable between the two groups, including mortality, stroke, myocardial infarction, life-threatening bleeding and pacemaker implantation before discharge. Thirty-day follow-up outcomes are presented in Supplementary Table S1. The rates of all-cause mortality, myocardial infarction, stroke, bleeding, and permanent pacemaker implantation also did not significantly differ between the two groups.

### 3.3. DW-MRI Findings After TAVR

All patients underwent high-quality DW-MRI. The findings for horizontal aorta and non-horizontal aorta patients are displayed in Table 3. The median interval from TAVR to DW-MRI examination was 2 days. Most patients presented with new-onset cerebral infarcts on DW-MRI imaging, with no significant difference between the two cohorts (89.9% vs. 83.3%; *p* = 0.157). Most cerebral infarcts were diffusely scattered, and multiple lesions were more common than single lesions (87.3% vs. 12.7%). Patients with horizontal aorta had a higher number of lesions detected by DW-MRI (4.0 [IQR: 2.0 to 9.0] vs. 3.0 [IQR: 1.0 to 7.0]; *p* = 0.031). A larger total volume of new lesions (320.0 mm^3^ [IQR: 80.0 to 915.0] vs. 180.0 mm^3^ (60.0–502.5); *p* = 0.017) and a larger volume per lesion (70.0 mm^3^ [IQR: 40.0 to 102.5] vs. 51.7 mm^3^ [IQR: 22.8 to 86.4]; *p* = 0.036) were also more frequently observed in the horizontal aorta group. The proportion of patients with a lesion size > 1 cm^3^ was higher in horizontal aorta patients (23.2% vs. 11.4%; *p* = 0.009). Of note, the incidence of cerebral infarctions in all territories was comparable across cohorts.

### 3.4. Predictors of Cerebral Ischemic Lesion Burden

Factors associated with the number of cerebral infarctions demonstrated in univariate and multivariate linear regression are shown in Table 4. In the multivariate linear regression model (forward stepwise method) adjusted for baseline variables, horizontal aorta (*p* = 0.007), along with older age (*p* = 0.002) and BAV (*p* = 0.036), was independently associated with a greater number of cerebral infarctions. When aortic angle was analyzed as a continuous variable in the entire cohort, a greater aortic angle was significantly associated with an increased number of cerebral ischemic lesions after TAVR (β = 0.103, *p* = 0.037).

## 4. Discussion

To the best of our knowledge, this is the first study focusing on the association between the aortic angle and cerebral infarctions following TAVR. Our study revealed several novel insights into this anatomical feature: (1) the overall incidence of periprocedural overt stroke was 2.7%, whereas new-onset cerebral infarctions detected by DW-MRI were present in 84.2% of patients; (2) No statistically significant differences were observed in periprocedural complications or 30-day clinical outcomes between the horizontal aorta and non-horizontal aorta groups; (3) patients with horizontal aorta had a higher number, a larger total volume and a larger volume per lesion of ischemic cerebral infarctions; and (4) Horizontal aorta, older age, and BAV were associated with an increased number of cerebral lesions after TAVR.

Importantly, the increased burden of DW-MRI-detected cerebral ischemic lesions in patients with horizontal aorta did not translate into a higher incidence of clinically overt stroke or worse 30-day clinical outcomes in the present study. This may be explained by the fact that most cerebral ischemic lesions detected after TAVR are clinically silent and do not result in immediate neurological symptoms. Nevertheless, these lesions should not be considered entirely benign. Previous studies have suggested that silent cerebral ischemic lesions detected by DW-MRI after TAVR are associated with subsequent neurocognitive decline, impaired physical performance, and reduced quality of life [8,9,10,11].

TAVR has revolutionized the treatment of symptomatic severe AS in the elderly and has proven superior to traditional traumatic SAVR [3,4]. However, certain anatomical conditions, such as BAV, an elliptic annulus, a heavily calcified annulus, and horizontal aorta, are present in clinical practice and may adversely affect prosthesis performance and counteract the clinical benefits of TAVR. Among these, horizontal aorta is of great concern due to its high incidence of 16.8% and warrants in-depth investigation [15]. Horizontal aorta is a dynamic anatomical feature that varies over time, possibly related to age and individual constitution [14]. Horizontal aorta, resulting in horizontal shift of the aortic axis, poses a technical challenge, particularly for the TAVR via transfemoral approach [17]. This unfavorable anatomical feature may complicate the delivery of the prosthesis by hindering the delivery system from passing through the diseased native valve smoothly. Concordantly, the snare technique was applied more frequently in horizontal aorta group, which was always used to achieve a more coaxial alignment by pulling the delivery system toward the center of the aortic curvature. The above-stated manipulations result in more interaction between the flexible delivery system, calcified native valve, and aortic wall during balloon valvuloplasty and valve deployment [16,18,19]. In addition to anatomical factors, several procedural factors may also contribute to cerebral embolization during TAVR. Procedural manipulations, including balloon valvuloplasty and device positioning and deployment, may increase mechanical interaction between the delivery system, calcified native valve, and aortic wall, thereby potentially promoting embolic debris release [19]. Operator experience and procedural technique may also influence embolic burden. Furthermore, cerebral embolic protection strategies may modify the risk of cerebral embolization; however, large randomized trials have not demonstrated a significant reduction in clinically overt periprocedural stroke with routine cerebral embolic protection (CEP) [11,20]. In the present study, these procedural factors were not systematically recorded or standardized, limiting our ability to further evaluate their potential contribution to cerebral ischemic lesion burden. The increases in both total fluoroscopy time (*p* = 0.010) and contrast dose (*p* = 0.085) were also manifestations of the greater procedural complexity in the horizontal aorta group. Similarly, patients with horizontal aorta presented with more pronounced aortic root dilation compared to those without horizontal aorta. It is worth noting that larger valve prostheses can hamper ideal positioning and coaxial alignment within the aortic annulus, potentially resulting in uneven stress on the aortic annulus and prosthesis deformation [11].

Nearly half of procedure-related cerebrovascular events occur within 24 h following TAVR, and they are closely related to intraoperative manipulations [21]. Fortunately, the risk of cerebrovascular events has declined in recent years, which can be attributed to the iteration of the prosthesis, accumulation of operators’ experience, and optimization of TAVR technology [1,2]. These cerebral infarctions were typically multiple (rather than solitary) and distributed across multiple vascular territories bilaterally, a pattern strongly suggestive of a cardiogenic embolic origin [19]. The shedding of atherosclerotic or calcified plaque, which is considered the major cause of cerebral embolism during TAVR, can result from various intraoperative steps such as intravascular instrument manipulation, balloon valvuloplasty, and prosthesis deployment [22,23].

Horizontal aorta has been identified as a strong determinant of an enhanced risk of cerebral ischemic infarctions after TAVR. Consistent with this, the incidence of new cerebral infarctions was numerically higher in the horizontal aorta group (89.9% vs. 83.3%; *p* = 0.157). Moreover, patients with horizontal aorta had a higher number of lesions detected by DW-MRI (4.0 [IQR: 2.0 to 9.0] vs. 3.0 [IQR: 1.0 to 7.0]; *p* = 0.031). These lesions were also more extensive in the horizontal aorta group, as evidenced by a larger total lesion volume (320.0 mm^3^ [IQR: 80.0–915.0] vs. 180.0 mm^3^ [IQR: 60.0–502.5]; *p* = 0.017) and a larger volume per lesion (70.0 mm^3^ [IQR: 40.0–102.5] vs. 51.7 mm^3^ [IQR: 22.8–86.4]; *p* = 0.036). Finally, a greater proportion of horizontal aorta patients had lesions larger than 1 cm^3^ (23.2% vs. 11.4%; *p* = 0.009).

It is well-recognized that elderly patients are prone to cerebrovascular events. Recent investigations have shown that the risk of postoperative stroke is more than three times higher in individuals over 80 years old than in those under 70 (2.5% vs. 0.7%) [24,25,26,27]. In our multivariate regression model, ageing was confirmed as a risk factor for cerebral infarction. This is interesting in the context of previous studies which have found a correlation between horizontal aorta and ageing [14], and Matsuda Y et al. found that ageing was an independent predictor for horizontal aorta. However, our study did not further explore the predictors of horizontal aorta [26]. We hypothesize that these findings may be explained by the dislodgement of micro-debris from age-related advanced atherosclerosis and calcification [28]. Concordant with the results of our previous study [29], BAV is an independent predictive factor for cerebral infarctions after TAVR.

There is evidence indicating that perioperative ischemia may increase the risk of long-term neurocognitive decline [30]. CEP devices represent a potential strategy for mitigating cerebral embolization during TAVR. However, large randomized trials have not demonstrated a significant reduction in clinically overt periprocedural stroke with routine CEP use [11,20]. Whether selected high-risk populations, such as patients with horizontal aorta and greater procedural complexity, may derive benefit from CEP remains uncertain and warrants further investigation [31,32].

This study has several limitations inherent to its observational, non-randomized design. First, to some extent, the generalizability of our findings may be limited because we included only patients who underwent TAVR via transfemoral approach. Moreover, because this single-center study exclusively included self-expanding prostheses, the applicability of our findings to balloon-expandable valves and other contemporary TAVR platforms remains uncertain. Second, the number of attempts at prosthesis positioning for new-generation devices and the frequency of balloon dilatation were not systematically recorded; therefore, their effect on cerebral ischemic events cannot be interpreted in the present study. Additionally, although patients with horizontal aorta accounted for 16.8% of the study population, the sample size of the horizontal aorta group was substantially smaller than that of the non-horizontal aorta group. Lastly, our study did not assess the association between MRI findings and neurocognitive outcomes. Future long-term follow-up studies are needed to better understand the clinical impact of this complication.

## 5. Conclusions

Horizontal aorta is a relatively uncommon but clinically relevant anatomical feature among TAVR candidates. Although TAVR-related clinical complications were comparable between patients with and without horizontal aorta, patients with horizontal aorta had a greater burden of DW-MRI-detected cerebral ischemic lesions, as reflected by both lesion number and lesion volume. This may be partly attributable to increased procedural complexity, more challenging coaxial prosthesis alignment, and greater device-valve interaction during TAVR.

## Figures and Tables

**Table 1:** Baseline clinical, echocardiographic, and computed tomographic characteristics.

	Overall *N* = 411	Horizontal Aorta > 60° *n* = 69	Non-Horizontal Aorta ≤ 60° *n* = 342	*p* Value
Age, yrs	74.0 (69.0–79.0)	75.0 (70.5–80.0)	74.0 (69.0–79.0)	0.161
Male	224 (54.5)	37 (53.6)	187 (54.7)	0.872
BMI, kg/m^2^	22.9 (20.4–25.1)	23.9 (21.7–26.6)	22.8 (20.2–25.0)	**0.006**
BAV	240 (58.4)	47 (68.1)	193 (56.4)	0.072
STS, %	3.83 (2.29–6.39)	3.47 (2.21–5.63)	3.89 (2.29–6.48)	0.520
NYHA III/IV	324 (78.8)	61 (88.4)	263 (76.9)	**0.033**
Smoker	87 (21.2)	13 (18.8)	74 (21.6)	0.604
Hypertension	219 (53.3)	47 (68.1)	172 (50.3)	**0.007**
Diabetes	87 (21.2)	10 (14.5)	77 (22.5)	0.137
Atrial fibrillation	58 (14.1)	13 (18.8)	45 (13.2)	0.216
CKD	202 (49.1)	30 (43.5)	172 (50.3)	0.302
PVD	25 (6.1)	4 (5.8)	21 (6.1)	1.000
Prior PCI	39 (9.5)	3 (4.3)	36 (10.5)	0.110
Prior stroke	19 (4.6)	4(5.8)	15 (4.4)	0.845
Prior MI	5 (1.2)	0 (0)	5 (1.2)	0.595
EF, %	60.6 (50.9–65.3)	62.1 (50.8–64.9)	62.1 (50.8–66.3)	0.225
EF ≤ 50%	100 (24.3)	17 (24.6)	83 (24.3)	0.948
Max velocity, m/s	4.75 (4.30–5.37)	4.48 (4.19–5.01)	4.79 (4.31–5.47)	**0.009**
Mean gradient, mmHg	52.0 (42.0–67.0)	57.0 (40.5–61.0)	53.0 (43.0–69.0)	**0.018**
AVA, cm^2^	0.60 (0.46–0.76)	0.59 (0.45–0.80)	0.67 (0.46–0.76)	0.205
≥moderate MR	113 (27.6)	18 (26.1)	95 (27.9)	0.753
≥moderate TR	54 (13.2)	11 (15.9)	43 (12.6)	0.461
Perimeter derived diameter, mm	24.3 (22.9–26.3)	24.4 (22.9–26.3)	24.3 (22.9–26.3)	0.838
STJ diameter, mm	30.3 (27.6–33.0)	33.0 (30.4–34.7)	29.8 (26.9–32.2)	**<0.001**
STJ height, mm	21.8 (19.5–24.7)	22.1 (19.7–25.2)	21.6 (19.1–23.6)	**0.007**
Ascending aorta diameter at 4 cm above the annulus, mm	37.4 (34.2–40.7)	40.0 (37.9–42.8)	36.6 (33.8–40.2)	**<0.001**
Aortic root angle, degrees	51.0 (45.0–57.0)	65.0 (62.0–69.0)	49.0 (43.0–53.0)	**<0.001**
LM height, mm	14.2 (12.2–16.7)	14.4 (12.6–17.2)	14.2 (12.0–16.6)	0.182
RCA height, mm	16.4 (14.4–18.5)	16.6 (15.1–19.4)	16.4 (14.4–18.2)	0.148
≥severe calcification	198 (48.1)	30 (43.5)	168 (49.0)	0.404

Values are presented as n (%), or median (interquartile range). *p* values in bold are statistically significant. BMI = body mass index; BAV = bicuspid aortic valve; STS = the Society of Thoracic Surgeons; NYHA = New York Heart Association; CKD = chronic kidney disease; PVD = peripheral vascular disease; PCI = percutaneous coronary intervention; MI = myocardial infarction; EF = ejection fraction; AVA = aortic valve area; MR = mitral regurgitation; TR= tricuspid regurgitation; STJ = sinotubular junction; LM = left main artery; RCA = right coronary artery.

**Table 2:** Procedural characteristics and in-hospital clinical outcomes.

	Overall *N* = 411	Horizontal Aorta > 60° *n* = 69	Non-Horizontal Aorta ≤ 60° *n* = 342	*p* Value
Procedural characteristics				
Pre-dilatation	400 (97.3)	68 (98.6)	332 (97.1)	0.699
Post-dilatation	274 (66.7)	45 (65.2)	229 (67.0)	0.780
Contrast, mL	110.0 (97.8–125.0)	113.0 (100.0–142.5)	110.0 (95.0–120.0)	0.085
Fluoroscopy time, min	23.6 (19.3–31.5)	26.3 (21.2–35.7)	23.3 (19.0–30.5)	**0.010**
Clinical outcomes				
Second transcatheter heart valve implantation	32 (7.8)	8 (11.6)	24 (7.0)	0.196
Conversion to SAVR	0 (0)	0 (0)	0 (0)	-
Coronary obstruction	1 (0.2)	1 (1.4)	0 (0)	0.168
Annular rupture	1 (0.2)	0 (0)	1 (0.3)	1.000
Aortic dissection	5 (1.2)	0 (0)	5 (1.5)	0.595
Mortality	1 (0.2)	1 (1.4)	0 (0)	0.168
Cardiovascular	1 (0.2)	1 (1.4)	0 (0)	0.168
Non-cardiovascular	0 (0)	0 (0)	0 (0)	-
Stroke	11 (2.7)	1 (1.4)	10 (2.9)	0.777
Disabling	5 (1.2)	0 (0)	5 (1.5)	0.595
Non-disabling	6 (1.5)	1 (1.4)	5 (1.5)	1.000
Myocardial infarction	1 (0.2)	1 (1.4)	0 (0)	0.168
Life-threatening bleeding	1 (0.2)	1 (1.4)	0 (0)	0.168
Pacemaker implantation	10 (2.4)	1 (1.4)	9 (2.6)	0.878

Values are presented as n (%), or median (interquartile range). *p* values in bold are statistically significant. SAVR = surgical aortic valve replacement; NYHA = New York Heart Association. Second valve implantation was defined as implantation of an additional transcatheter heart valve during the index TAVR procedure because of suboptimal positioning, valve migration, or unsatisfactory prosthetic valve function after deployment.

**Table 3:** DW-MRI Findings of horizontal aorta and non-horizontal aorta patients.

	Overall *N* = 411	Horizontal Aorta > 60° *n* = 69	No-Horizontal Aorta ≤ 60° *n* = 342	*p*
Time from TAVR to DW-MRI examination, days	2.0 (1.0–4.0)	3.0 (1.0–5.0)	2.0 (1.0–4.0)	0.038
Patients with new lesions	346 (84.2)	62 (89.9)	284 (83.0)	0.157
Total new lesions	2208	523	1685	-
New lesions per patients	3.0 (1.0–7.0)	4.0 (2.0–9.0)	3.0 (1.0–7.0)	0.031
Patients with a single lesion	44 (12.7)	9 (14.5)	35 (12.3)	0.639
Patients with multiple lesions	302 (87.3)	53 (85.5)	249 (87.7)	-
Patients with bi-hemispheric lesions	231 (66.8)	44 (71.0)	187 (65.8)	0.438
Lesion location				
ACA	113 (27.5)	24 (34.8)	89 (26.0)	0.137
ACA/MCA	170 (41.4)	32 (46.4)	138 (40.4)	0.354
MCA	220 (53.5)	44 (63.8)	176 (51.5)	0.062
MCA/PCA	52(12.7)	6 (8.7)	46 (13.5)	0.278
PCA	190 (46.2)	37 (53.6)	153 (44.7)	0.177
VA/BA	224 (54.5)	42 (60.9)	182 (53.2)	0.244
Lesion volume, mm^3^, per lesion	55.0 (26.0–88.0)	70.0 (40.0–102.5)	51.7 (22.8–86.4)	0.036
Maximal lesion volume, mm^3^, per patient	90.0 (40.0–200.0)	110.0 (60.0–235.0)	90.0 (40.0–190.0)	0.100
Total lesion volume, mm^3^, per patient	190.0 (60.0–570.0)	320.0 (80.0–915.0)	180.0 (60.0–502.5)	0.017
Total lesion volume ≥ 1000 mm^3^	55 (13.4)	16 (23.2)	39 (11.4)	0.009

Data are presented as n (%), mean ± standard deviation, or median (interquartile range), as appropriate. DW-MRI = diffusion-weighted magnetic resonance imaging; ACA = anterior cerebral artery; MCA = middle cerebral artery; PCA = posterior cerebral artery; VA = vertebral artery; BA = basilar artery.

**Table 4:** Linear model results for predictors of the number of cerebral infarctions.

Variables	The Number of the Lesions			
	Univariate		Multivariate	
	β (SE)	*p* Value	β (SE)	*p* Value
Age, yrs	0.146	0.003	0.158	0.002
BAV	0.093	0.060	0.105	0.036
Male	−0.062	0.210	-	-
BMI, kg/m^2^	−0.075	0.129	-	-
STS, %	0.017	0.729	-	-
NYHA III/IV	−0.044	0.371	-	-
CKD	0.101	0.041	0.053	0.337
Hypertension	−0.025	0.607	-	-
Diabetes	−0.053	0.281	-	-
PVD	−0.002	0.967	-	-
Prior stroke	0.013	0.800		
EF ≤ 50%	0.083	0.093	0.067	0.172
Max velocity, m/s	−0.032	0.520		
AVA, cm^2^	−0.114	0.022	−0.079	0.126
≥moderate MR	−0.001	0.984	-	-
≥moderate TR	−0.024	0.624	-	-
Perimeter derived diameter, mm	−0.002	0.963	-	-
STJ diameter ≥ 31.0 mm	0.049	0.318	-	-
STJ height, mm	−0.001	0.986	-	-
Ascending aorta diameter at 4 cm, mm	0.126	0.011	-	-
Ascending aorta diameter ≥ 40 mm (at 4 cm)	0.111	0.024	0.071	0.161
Horizontal aorta	0.155	0.002	0.132	0.007
≥severe calcification	−0.022	0.664	-	-

*p* value <0.10 in the univariate analysis were entered into the multivariate model with stepwise forward method. Abbreviations: BAV = bicuspid aortic valve; BMI = body mass index; STS = the Society of Thoracic Surgeons; NYHA = New York Heart Association; CKD = chronic kidney disease; PVD = peripheral vascular disease; EF = ejection fraction; AVA = aortic valve area; MR = mitral regurgitation; TR= tricuspid regurgitation; STJ = sinotubular junction.

## Data Availability

The anonymized data underlying this article are available from the corresponding author upon reasonable request, subject to approval by the Second Affiliated Hospital, Zhejiang University School of Medicine.

## Supplementary Materials

The following supporting information can be downloaded at: https://www.mdpi.com/article/10.3390/jcm15176715/s1, Table S1. Thirty-day clinical outcomes.

## Abbreviations

TAVR	transcatheter aortic valve replacement
HA	horizontal aorta
DW-MRI	diffusion-weighted magnetic resonance imaging
AS	aortic stenosis
IQR	interquartile range
STS	The Society of Thoracic Surgeons
SAVR	surgical aortic valve replacement
MDCT	multidetector computed tomography
ADC	apparent diffusion coefficient
BAV	bicuspid aortic valve
