# Supplementary material for: Cerebral Infarctions After Transcatheter Aortic Valve Replacement of Horizontal Aorta Versus Non-Horizontal Aorta
Source: J Clin Med. 2026 Aug 29;15(17):6715. doi: 10.3390/jcm15176715 (PMC13566855; doi:10.3390/jcm15176715)
Supplement: Supplementary file 1 [file jcm-15-06715-s001.zip › jcm-4430974-supplementary.pdf]

Supplementary Table S1. 30-day clinical outcomes

	Overall N=411	Horizontal aorta > 60° n=69	Non-Horizontal aorta ≤ 60° n=342	P value
Mortality	3 (0.7)	2 (2.9)	1 (0.3)	0.074
Cardiovascular	3 (0.7)	2 (2.9)	1 (0.3)	0.074
Non-cardiovascular	0 (0)	0 (0)	0 (0)	-
NYHA III/IV	24 (5.9)	4 (6.0)	20 (5.9)	1.000
Myocardial infarction	1 (0.2)	1 (1.4)	0 (0)	0.169
Life-threatening bleeding	1 (0.2)	1 (1.4)	0 (0)	0.168
Pacemaker implantation	15 (3.7)	1 (1.4)	14 (4.1)	0.469

Values are presented as n (%), or median (interquartile range). P values in bold are statistically significant.

SAVR=surgical aortic valve replacement; NYHA = New York Heart Association.
